# Maternal Grand Multiparity and the Risk of Severe Mental Disorders in Adult Offspring

**DOI:** 10.1371/journal.pone.0114679

**Published:** 2014-12-10

**Authors:** Marius Lahti, Johan G. Eriksson, Kati Heinonen, Eero Kajantie, Jari Lahti, Kristian Wahlbeck, Soile Tuovinen, Anu-Katriina Pesonen, Maiju Mikkonen, Clive Osmond, Katri Räikkönen

**Affiliations:** 1 Institute of Behavioural Sciences, University of Helsinki, Helsinki, Finland; 2 National Institute for Health and Welfare, Diabetes Prevention Unit, Helsinki, Finland; 3 Institute of Clinical Medicine, University of Helsinki, Helsinki, Finland; 4 Vaasa Central Hospital, Vaasa, Finland; 5 Unit of General Practice, Helsinki University Central Hospital, Helsinki, Finland; 6 Folkhälsan Research Centre, Helsinki, Finland; 7 Children’s Hospital, Helsinki University Central Hospital and University of Helsinki, Helsinki, Finland; 8 Department of Obstetrics and Gynaecology, MRC Oulu, Oulu University Hospital and University of Oulu, Oulu, Finland; 9 The Finnish Association for Mental Health, Helsinki, Finland; 10 MRC Lifecourse Epidemiology Unit, University of Southampton, Southampton, United Kingdom; Leibniz Institute for Neurobiology, Germany

## Abstract

**Background:**

Previous studies have shown that maternal grand multiparity may predict an increased risk of mental disorders in young adult offspring, but whether such effects persist throughout adulthood remains unknown. The current study examined if maternal grand multiparity predicts the risks of severe mental disorders, suicides, suicide attempts and dementias throughout adult life.

**Methods:**

Our study sample comprised 13243 Helsinki Birth Cohort Study 1934–1944 participants (6905 men and 6338 women). According to hospital birth records, 341 offspring were born to grand multiparous mothers. From Finnish national hospital discharge and causes of death registers, we identified 1682 participants diagnosed with mental disorders during 1969–2010.

**Results:**

Maternal grand multiparity predicted significantly increased risks of mood disorders (Hazard Ratio = 1.64, p = 0.03), non-psychotic mood disorders (Hazard Ratio = 2.02, p = 0.002), and suicide attempts (Hazard Ratio = 3.94, p = 0.01) in adult offspring. Furthermore, women born to grand multiparous mothers had significantly increased risks of any severe mental disorder (Hazard Ratio = 1.79, p = 0.01), non-psychotic substance use disorders (Hazard Ratio = 2.77, p = 0.02) schizophrenia, schizotypal and delusional disorders (Hazard Ratio = 2.40, p = 0.02), mood disorders (Hazard Ratio = 2.40, p = 0.002), non-psychotic mood disorders (Hazard Ratio = 2.91, p<0.001), and suicide attempts (Hazard Ratio = 5.05, p = 0.01) in adulthood. The effects of maternal grand multiparity on offspring psychopathology risk were independent of maternal age and body mass index at childbirth, and of year of birth, sex, childhood socioeconomic position, and birth weight of the offspring. In contrast, no significant effects were found among men.

**Conclusions:**

Women born to grand multiparous mothers are at an increased risk of severe mental disorders and suicide attempts across adulthood. Our findings may inform the development of preventive interventions for mental disorders.

## Introduction

A few previous studies have shown that maternal grand multiparity (birth from sixth or later pregnancy lasting more than 20 weeks) may predict an increased risk of psychopathology in adult offspring [1]–[3]. These studies have shown that among offspring born to grand multiparous mothers increased risks are evident for alcoholism [2], mood disorders [2], schizophrenia [3], and other psychotic disorders [2], and suicides [1]. On the other hand, other studies have shown that offspring born as a fourth or later born -child have higher risks of psychiatric hospitalizations for any reason, [4] and of suicides [4]–[5] and suicide attempts [4], [6] and that offspring born as a third or later-born child have an increased risk of personality disorders [7] in adult life.

However, the existing studies on maternal grand multiparity and mental disorders have mostly followed up the offspring only until young adulthood [1]–[3]. To our knowledge, it remains unknown if the risks associated with maternal grand multiparity for mental disorders in the offspring persist after the transition into adulthood and to a more independent role in the society. It is also uncertain if maternal grand multiparity is independently associated with the risk of mental disorders or whether the associations found are confounded by maternal age at childbirth; a known correlate of parity and psychopathology risk in the offspring [1], [3]–[9]. Furthermore, although maternal grand multiparity has been shown to predict suicide risk particularly among men [1], the other studies on grand multiparity and offspring psychopathology risk have not examined whether the associations differ by sex [2]–[3].

Hence, in this longitudinal study, we examined the associations between maternal grand multiparity and offspring risks of mental disorders severe enough to warrant hospitalization or contribute to death throughout adulthood, from 1969 when the participants were between 24 and 34 years of age to 2010, when the participants were between 66 and 76 years of age. We also examined the associations to suicides and suicide attempts, and to organic dementias. We also studied whether the potential risks associated with maternal grand multiparity were independent of maternal age at childbirth, and whether the associations differed by sex.

## Materials and Methods

### Study population

**Figure 1:**
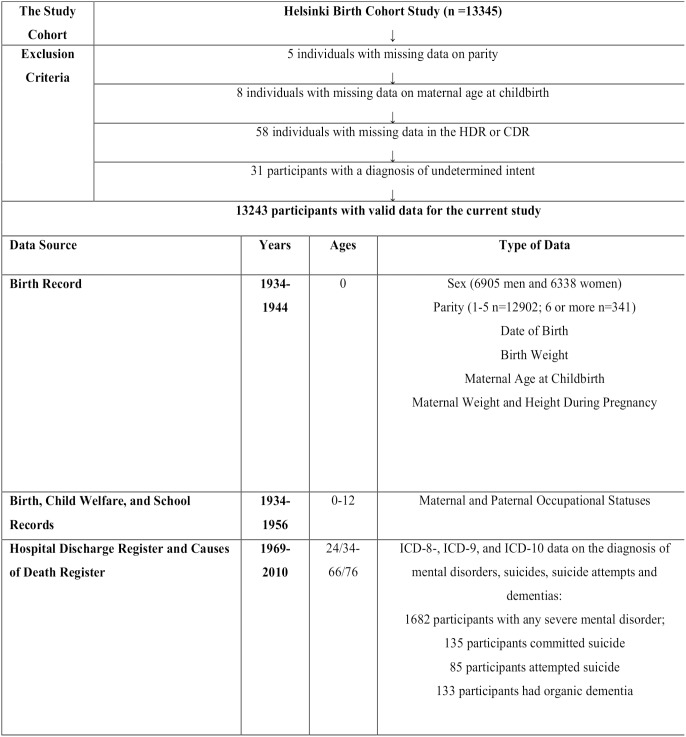
The participants, the exclusion criteria and the data sources for the current study.

The current study was conducted among the Helsinki Birth Cohort Study (HBCS) participants. The HBCS has been approved by the Ethics Committee of the National Public Health Institute. The HBCS comprises 13345 singleton live births (6975 men (52.3%) and 6370 women (47.7%)) at the two public maternity hospitals in Helsinki, Finland, between 1934 and 1944. From the current study we excluded five individuals with missing data on parity and eight individuals with missing data on maternal age at childbirth. There were 58 cohort members who were excluded due to missing data either in the Finnish Hospital Discharge Register (HDR) or in the Causes of Death Register (CDR). Also 31 individuals with a diagnosis of injury of undetermined intent or of usually self-inflicted poisoning were excluded (Fig. 1).

The available study sample thus comprised 13243 individuals, of whom 6905 (52.1%) were men and 6338 (47.9%) were women. Compared to the included participants, the 102 excluded cohort members were more often men (68.6% vs. 52.1%, p = .001) and came more frequently from families where the father was a manual worker (71.6 vs. 57.0%, p = 0.01).

### Maternal grand multiparity

Data on parity was extracted from hospital birth records (Fig. 1). Grand multiparity was defined as being born as sixth or later born child.

### Covariates and confounders

Also data on maternal age at childbirth was extracted from birth records (Fig. 1). Maternal age at childbirth was classified into four categories; <20 years, 20–29 years, 30–39 years, ≥40 years. Other covariates and confounders included sex, year of birth, and birth weight of the offspring, and maternal body mass index at childbirth (BMI), all extracted from hospital birth records, and socioeconomic position in childhood, data on which was attained from birth, child welfare, and school health records. Socioeconomic position in childhood and birth weight were examined as possible confounders, since both factors have been associated with maternal grand multiparity [2], [10]–[11] and with the risks of mental disorders [4], [9]. On the other hand, maternal BMI was used as a covariate to include a crude proxy measure of exposure to gestational diabetes, which is associated with both grand multiparity [12]–[13] and offspring risk of mental disorders [14].

Of birth weight, we used sex-standardized z-scores in all the analyses. Maternal BMI at childbirth was calculated as maternal weight divided by height squared (kg/m^2^). There were 1312 mothers with missing data on weight or on height. In these cases, we imputed the mean value of maternal BMI of the parity group the mother belonged to. Socioeconomic position in childhood was defined with two variables; one indexing the father’s and the other the mother’s highest attained occupational status. These variables were categorized into three categories: clerical workers, manual workers, and those with other occupations or missing occupational data. Missing maternal occupational data included the children of housewives and missing paternal data included children born to unmarried mothers. Although maternal and paternal occupational statuses were strongly interrelated (χ^2^(4) = 1650.6, p*<*0.001), in 40.1% of the families the parents belonged to different occupational categories.

### Outcome variables: mental disorders, suicides, suicide attempts, and organic dementias

We identified the diagnoses of mental disorders from the HDR and the CDR between 1.1.1969 (when the participants were between 24 and 34 years of age) and 31.12.2010 (when the participants were between 66 and 76 years old). The HDR carries the primary and up to three subsidiary discharge diagnoses of all hospitalizations and the CDR the primary, underlying, and contributory causes of death for all deaths in Finland. Both registers have been in use since 1969 when a personal identification number was given to each Finnish resident in Finland. In Finland, International Classification of Diseases, eighth revision (ICD-8) was used in clinical practice between 1969 and 1986; ICD-9, following the Diagnostic and Statistical Manual of Mental Disorders, Third Revision was used between 1987 and 1995; and ICD-10 has been in use since 1996. The HDR [15]–[16] and the CDR [17] are valid and reliable research tools. The HDR diagnoses of schizophrenia [18], bipolar disorder [19], any psychotic disorder [20] and dementias [21] each shows high specificity. Also the validity of the HDR ICD-8 diagnoses of alcohol dependence and alcohol psychosis [15], [22] and ICD-9 and ICD-10 depressive disorders [23] have gained some research support.

A diagnosis of an injury of undetermined intent may or may not represent suicide [24]. Hence, we excluded from this study individuals with a diagnosis of injury of undetermined intent or poisoning of usually self-inflicted intent with no co-occurring diagnosis of mental disorders. The diagnostic codes used to identify these exclusion diagnoses were E98 from ICD-8, E97 from ICD-9, and Y10–Y34, T39, and T42–T43 from ICD-10.

As diagnostic outcomes, we identified any mental disorder severe enough to require hospitalization or contribute to death (referred to hereafter as any severe mental disorder), and the specific diagnostic categories of substance use disorders, schizophrenia, schizotypal, and delusional disorders, mood disorders, anxiety disorders, and personality disorders. We also examined associations to the subcategories of psychotic and non-psychotic substance use and mood disorders. In these analyses, a diagnosis of psychotic substance use or mood disorder was considered primary and was used as exclusion criteria for non-psychotic disorders. Furthermore, as mentioned, we studied the associations between maternal grand multiparity and suicides, suicide attempts, and organic dementias. The diagnostic codes corresponding to all these diagnostic categories are shown in Table 1, together with the number and the percentage of participants with each diagnosis and the median age at first hospitalization or death with each diagnosis. Between 1969 and 2010, there were 1682 participants (12.7%; 1059 men and 623 women) who had been hospitalized with a diagnosis of mental disorder as a hospital discharge diagnosis or who had died with a diagnosis of mental disorder included in the death certificate. These 1682 participants had received their first diagnosis of mental disorder at a median age of 43.4 years (range 19.0–76.6 years). Furthermore, there were 135 (1.0) participants who had committed suicide, 85 (0.6) participants who had attempted suicide, and 133 (1.0%) participants who had a diagnosis of organic dementia. In all the analyses, the control outcome group was composed of all participants with no mental disorder, suicide, suicide attempt, or organic dementia diagnoses. Thus, the 11404 participants (5746 men and 5658 women) who had no diagnosis of mental disorder, suicide, suicide attempt, or organic dementia always served as the comparison outcome group.

**Table 1:** The Cumulative Incidence of Any Mental Disorder, Specific Mental Disorders, Suicides, Suicide Attempts and Organic Dementias and Median Age at First Diagnosis.

		All Participants		Women	Men	
Diagnostic Category	ICD DiagnosticCodes	CumulativeIncidence (%)	Median Age(Years) at FirstDiagnosis (Range)	CumulativeIncidence (%)	CumulativeIncidence (%)	p^a^
Any Mental Disorder b	**ICD-8:** 291, 295,296–305, 306.4–306.5,306.8, 306.98, 307 **ICD-9:**291–292, 295–298,300–304, 305, 3071A,3074, 3075A–3075B,3078A, 3079X, 3090A,3092C–3099X, 312**ICD-10:** F1–F6	1682 (12.7%)	43.4 (19.0–76.6)	623 (9.8%)	1059 (15.3%)	<0.001
Substance Use Disorders c	**ICD-8:** 291, 303–304**ICD-9:** 291–292,303–305 **ICD-10:**F10–F19	924 (7.0%)	45.9 (25.5–76.6)	193 (3.0%)	731 (10.6%)	<0.001
Psychotic Substance Use Disorders	**ICD-8:** 291 **ICD-9:**291–292; **ICD-10:**F1×5	293 (2.2%)	50.0 (27.2–75.5)	59 (0.9%)	234 (3.4%)	<0.001
Non-Psychotic Substance Use Disorders	**ICD-8:** 303–304**ICD-9:** 303–305**ICD-10:** F1×0-F1×4,F1×6-F1×9	631 (4.8%)	46.0 (25.8–76.6)	134 (2.1%)	497 (7.2%)	<0.001
Schizophrenia, Schizotypal, and Delusional Disorders	**ICD-8:** 295, 297,298.10–299.99 **ICD-9:**295, 297–298 **ICD-10:**F20-F29	345 (2.6%)	38.8 (19.0–73.6)	163 (2.6%)	182 (2.6%)	0.43
Mood Disorders	**ICD-8:** 296, 298.00,3004, 301.10 **ICD-9:**296, 3004A, 3011D**ICD-10:** F30–F39	622 (4.7%)	48.1 (24.5–75.6)	312 (4.9%)	310 (4.5%)	0.76
Psychotic Mood Disorders	**ICD-8:** 296, 298.00**ICD-9:** 2961E,2962–2963, 2967A**ICD-10:** F30–F31,F323, F333	145 (1.1%)	52.1 (24.5–75.2)	74 (1.2%)	71 (1.0%)	0.74
Non-Psychotic Mood Disorders	**ICD-8:** 3004, 301.10**ICD-9:** 2961A–2961D,2961F–2961G, 2968A,3004A, 3011D**ICD-10:** F320–F322,F328–F329,F330–F332, F334–F339,F34-F39	477 (3.6%)	48.1 (26.1–75.6)	238 (3.8%)	239 (3.5%)	0.88
Anxiety Disorders	**ICD-8:** 300.00–300.30,300.50–300.99, 305,306.80, 307.99 **ICD-9:**3000A–3003A,3006A–3009X, 3078A,309 **ICD-10:** F40-F48	332 (2.5%)	40.0 (25.6–71.0)	166 (2.6%)	166 (2.4%)	0.80
Personality Disorders	**ICD-8:** 301.00,301.20–301.99 **ICD-9:**3010A, 3012A–3015A,3016A–3018X **ICD-10:**F60-F61	209 (1.6%)	41.5 (24.8–66.6)	92 (1.5%)	117 (1.7%)	0.14
Suicides	**ICD-8 & ICD-9:**E95 **ICD-10:** R45.8,X60-X84; Y87.0	135 (1.0%)	48.8 (29.0–70.5)	30 (0.5%)	105 (1.5%)	<0.001
Suicide Attempts	**ICD-8:** E95	85 (0.6%)	38.4 (19.6–49.4)	45 (0.7%)	40 (0.6%)	0.47
Organic Dementias	**ICD-8:** 290.00–290.10**ICD-9:** 290, 2912A,2928C, 2941A, 3310A;3311A, 4378A **ICD-10:**F00, F01, F03, F051, G30	133 (1.0%)	66.6 (38.9–76.4)	60 (0.9%)	73 (1.1%)	0.15

a P-value indicating the statistical significance of the sex difference in the cumulative incidence of mental disorders.

b The diagnoses corresponding to the codes F50–F59 or F62–F69 in ICD-10 were included in the Any mental disorder –category, but were not assessed as a separate category.

c For the diagnoses of substance intoxifications (ICD-9; 305I; CD-10: F1x.0, only the primary diagnoses from the HDR and the CDR were included in the diagnostic categories. All other diagnostic entities include primary and up to three subsidiary hospital discharge diagnoses, and primary, underlying and contributory causes of death.

While in a longitudinal study such as this where a part of the cohort members have already passed away obtaining written consent to use the patient information included in the registers is impossible, we took several steps to anonymize the data in order to protect the privacy of the cohort members. First, the researchers sent a request to a data manager in charge of the HDR and CDR data usage to extract the diagnostic data from these two registers for the study participants, using the participants’ study identification codes as identification tools for whom to extract data. Thereafter, the data manager linked the study identification codes with the personal identification numbers of Finnish citizens, and sent a request to Statistics Finland to obtain the diagnostic data from these two registers for the cohort members. Once the data was received, the data manager anonymized the diagnostic information data, and sent the diagnostic data to the researchers of the current study with only the study identification codes as identification tools. The researchers conducting the study were unaware of the official personal identification numbers of the participants.

### Statistical analyses

We examined the associations of the covariates and confounders (sex, year of birth, maternal and paternal occupational statuses, maternal age and BMI at childbirth, and birth weight of the offspring) with maternal grand multiparity and with mental disorders in the offspring by using χ^2^- and t-tests and Cox Proportional Hazards models, respectively.

Then, we examined the effects of maternal grand multiparity on mental disorders with Cox Proportional Hazards models. The participants were followed up either until their first hospitalization for mental disorders, death, migration, or until December, 31, 2010. Since suicide attempts were coded into the HDR only during the use of ICD-8, in the analyses on suicide attempts the participants were followed up at longest until December, 31, 1986. All the analyses were stratified by year of birth and sex, and adjusted for mothers’ and father’s occupational statuses in childhood, the birth weight of the offspring, and maternal BMI at childbirth. In second models, maternal age at childbirth was also entered to the Cox Regression equation. All the analyses were repeated separately for men and women, and in separate Cox models, we examined interactions between maternal grand multiparity and sex on the risks of severe mental disorders, suicides, and suicide attempts, and organic dementias.

## Results

Table 2 shows the birth and the sociodemographic characteristics of the study participants by parity status. Individuals born in the earlier years, between 1934 and 1938, were more often born to grand multiparous mothers than individuals born between 1939 and 1944. Grand multiparous mothers had higher BMI at childbirth than mothers with lower parity and their offspring weighed more than the offspring born from earlier pregnancies. Grand multiparous births were more common among older mothers and among manual worker mothers, and in the families with manual worker fathers.

**Table 2:** The Birth and the Sociodemographic Characteristics of the Study Sample by Parity Status.

	Parity		
Characteristic	1–5 (n = 12902)	6 or more (n = 341)	
	Mean (SD^a^)/N (%)	Mean (SD^a^)/N (%)	p
Maternal Age at Childbirth	28.2 (5.3)	36.2 (4.7)	<0.001
Categorical:			<0.001
<20 years	338 (2.6%)	0 (0%)	
20 to 29 years	7626 (59.1%)	28 (8.2%)	
30 to 39 years	4622 (35.8%)	223 (65.4%)	
≥40 years	316 (2.4%)	90 (26.4%)	
Birth Weight (Grams)	3399.4 (475.7)	3694.3 (513.1)	<0.001
Maternal Body Mass Index (kg/m^2^)	26.2 (2.8)	27.6 (3.6)	<0.001
Fathers’ Occupational Status in Childhood			
Manual Worker	7293 (56.5%)	254 (74.5%)	<0.001
Clerical Worker	5264 (40.8%)	85 (24.9%)	
Unknown	345 (2.7%)	2 (0.6%)	
Mothers’ Occupational Status in Childhood			
Manual Worker	6592 (51.1%)	211 (61.9%)	<0.001
Clerical Worker	4942 (38.3%)	56 (16.4%)	
Other or Unknown	1368 (10.6%)	74 (21.7%)	
Year of Birth			
1934–1938	2930 (22.7%)	99 (29.0%)	0.01
1939–1944	9972 (77.3%)	242 (71.0%)	
Sex			
Women	6177 (47.9%)	161 (47.2%)	0.81
Men	6725 (52.1%)	180 (52.8%)	

a SD = Standard Deviation.

Men had higher risks of any severe mental disorder (HR = 1.61, p<0.001), substance use disorders (HR = 3.57, p<0.001), both psychotic (HR = 3.86, p<0.001), and non-psychotic (HR = 3.55, p<0.001) substance use disorders, and of suicides (HR = 3.40, p<0.001) than women (Table 1). Individuals born between 1939 and 1944 had higher risks of any severe mental disorder (HR = 1.19, p = 0.005), substance use disorders (HR = 1.21, p = 0.02), psychotic substance use disorders (HR = 1.92, p<0.001), mood disorders (HR = 1.29, p = 0.01), non-psychotic mood disorders (HR = 1.27, p = 0.04), anxiety disorders (HR = 1.44, p = 0.01), and personality disorders (HR = 2.01, p = 0.001) than individuals born between 1934 and 1938. As reported previously [25], the offspring of manual worker fathers had higher risks of any severe mental disorder (HR = 1.12, p = 0.03), substance use disorders (HR = 1.20, p = 0.01), non-psychotic substance use disorders (HR = 1.18, p = 0.05), and organic dementias (HR = 1.56, p = 0.02) than the offspring of clerical worker fathers. A smaller maternal BMI at childbirth predicted an increased risk of suicide attempts (HR = 0.92, p = 0.05). Maternal occupational status (p -values≥0.06), maternal age at childbirth (p-values≥0.07) or the birth weight of the offspring (p-values≥0.14) was not significantly associated with the risks of mental disorders, suicides, suicide attempts or organic dementias in the offspring.

### Maternal grand multiparity and mental disorders, suicides, suicide attempts, and organic dementias

**Figure 2:**
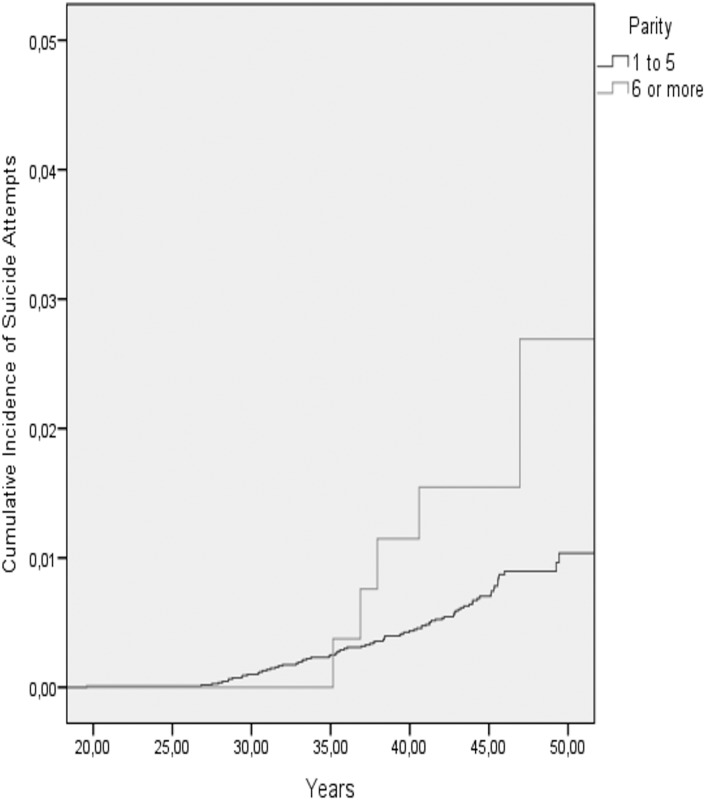
The cumulative incidence of suicide attempts among the offspring of non-grand multiparous and grand multiparous mothers.

**Figure 3:**
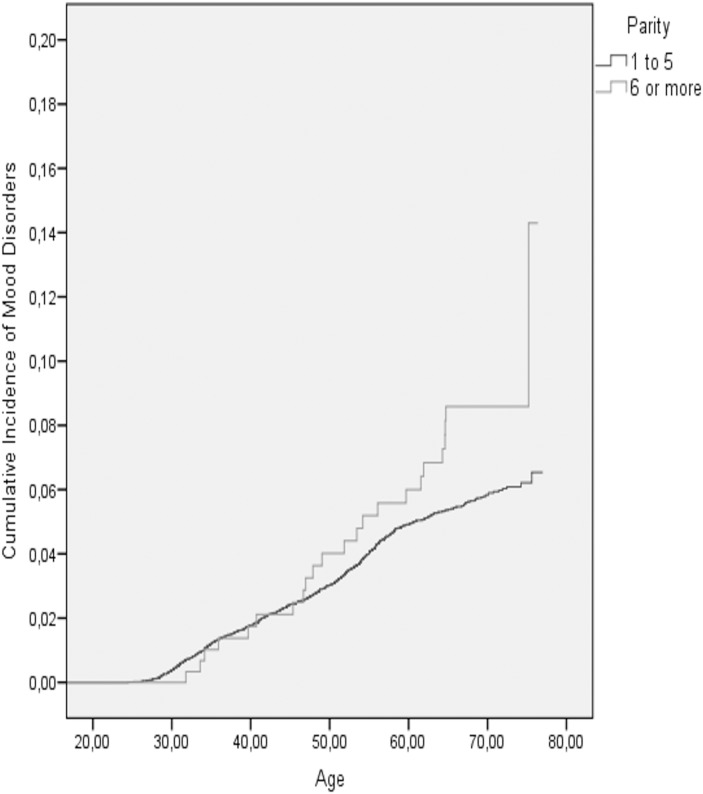
The cumulative incidence of mood disorders among the offspring of non-grand multiparous and grand multiparous mothers.

Table 3 shows the cumulative incidence of mental disorder, suicides, suicide attempts and organic dementias in the two parity groups, and Table 4 shows the results of the Cox Regression analyses on maternal grand multiparity and the aforementioned outcome variables. We found that in the models adjusted for the year of birth, sex, birth weight, maternal BMI, and maternal and paternal occupational statuses in childhood, maternal grand multiparity predicted a significantly increased, over 1.8 -fold risk of non-psychotic mood disorders (Table 4) and an over 3.3-fold risk of suicide attempts in adult offspring (Table 4; Fig. 2). In the models adjusted additionally for maternal age at childbirth, both of these associations remained significant, and the previously marginally significant association between maternal grand multiparity and an over 1.5-fold increased risk of any mood disorder in the offspring also became statistically significant (Tables 4; Fig. 3). In contrast, among all participants, maternal grand multiparity was not significantly associated with the risks of any severe mental disorder, substance use disorders, schizophrenia, schizotypal or delusional disorders, anxiety or personality disorders, committed suicides or organic dementias (Table 4).

**Table 3:** The cumulative incidence of mental disorders, suicides and suicide attempts by parity status.

	All Participants		Women		Men	
	Parity		Parity		Parity	
	1 to 5	6 or more	1 to 5	6 or more	1 to 5	6 or more
	N = 12902	N = 341	N = 6177	N = 161	N = 6725	N = 180
Diagnostic Outcome	N (%)	N (%)	N (%)	N (%)	N (%)	N (%)
Any Mental Disorder	1634 (12.7%)	48 (14.1%)	599 (9.7%)	24 (14.9%)	1035 (15.4%)	24 (13.3%)
Substance Use Disorders	899 (7.0%)	25 (7.3%)	185 (3.0%)	8 (5.0%)	714 (10.6%)	17 (9.4%)
Psychotic Substance Use Disorders	287 (2.2%)	6 (1.8%)	58 (0.9%)	1 (0.6%)	229 (3.4%)	5 (2.8%)
Non-Psychotic Substance Use Disorders	612 (4.7%)	19 (5.6%)	127 (2.1%)	7 (4.3%)	485 (7.2%)	12 (6.7%)
Schizophrenia, Schizotypal, and Delusional Disorders	333 (2.6%)	12 (3.5%)	155 (2.5%)	8 (5.0%)	178 (2.6%)	4 (2.2%)
Mood Disorders	599 (4.6%)	23 (6.7%)	298 (4.8%)	14 (8.7%)	301 (4.5%)	9 (5.0%)
Psychotic Mood Disorders	144 (1.1%)	1 (0.3%)	73 (1.2%)	1 (0.6%)	71 (1.1%)	0 (0.0%)
Non-Psychotic Mood Disorders	455 (3.5%)	22 (6.5%)	225 (3.6%)	13 (8.1%)	230 (3.4%)	9 (5.0%)
Anxiety Disorders	327 (2.5%)	5 (1.5)	164 (2.7%)	2 (1.2%)	163 (2.4%)	3 (1.7%)
Personality Disorders	204 (1.6%)	5 (1.5%)	90 (1.5%)	2 (1.2%)	114 (1.7%)	3 (1.7%)
Suicides	134 (1.0%)	1 (0.3%)	30 (0.5%)	0 (0.0%)	104 (1.5)	1 (0.6%)
Suicide Attempts	80 (0.6%)	5 (1.5%)	42 (0.7%)	3 (1.9%)	38 (0.6%)	2 (1.1%)
Organic Dementias	130 (1.0%)	3 (0.9%)	59 (1.0%)	1 (0.6%)	71 (1.1%)	2 (1.1%)

**Table 4:** Maternal Grand Multiparity and Severe Mental Disorders in the Offspring.

	All Participants		Women		Men	
Diagnostic outcome	HR^a^ (95% CI)^b^	p	HR^a^ (95% CI)^b^	p	HR^a^ (95% CI)^b^	p
Any Mental Disorder						
Model 1^c^	1.13 (0.84–1.51)	0.42	**1.74 (1.15–2.64)**	**0.01**	0.83 (0.55–1.25)	0.37
Model 2^d^	1.17 (0.87–1.58)	0.30	**1.79 (1.16–2.77)**	**0.01**	0.87 (0.57–1.32)	0.50
Substance Use Disorders						
Model 1^c^	1.07 (0.71–1.60)	0.76	2.00 (0.97–4.13)	0.06	0.86 (0.53–1.40)	0.54
Model 2^d^	1.15 (0.76–1.73)	0.52	2.07 (0.97–4.41)	0.06	0.94 (0.57–1.54)	0.80
Psychotic Substance Use Disorders						
Model 1^c^	0.75 (0.33–1.71)	0.50	0.80 (0.11–5.85)	0.83	0.73 (0.30–1.79)	0.49
Model 2^d^	0.81 (0.35–1.85)	0.61	0.75 (0.10–5.70)	0.78	0.81 (0.32–2.01)	0.64
Non-Psychotic Substance Use Disorders						
Model 1^c^	1.22 (0.77–1.94)	0.40	**2.56 (1.17–5.60)**	**0.02**	0.92 (0.51–1.64)	0.78
Model 2^d^	1.33 (0.82–2.14)	0.24	**2.77 (1.21–6.34)**	**0.02**	1.01 (0.56–1.82)	0.98
Schizophrenia, Schizotypal, and Delusional Disorders						
Model 1^c^	1.44 (0.80–2.59)	0.22	**2.43 (1.17–5.03)**	**0.02**	0.81 (0.30–2.22)	0.68
Model 2^d^	1.49 (0.81–2.73)	0.20	**2.40 (1.12–5.16)**	**0.02**	0.87 (0.31–2.42)	0.79
Mood Disorders						
Model 1^c^	1.51 (0.99–2.32)	0.06	**2.04 (1.18–3.52)**	**0.01**	1.07 (0.54–2.10)	0.84
Model 2^d^	**1.64 (1.06–2.54)**	**0.03**	**2.40 (1.36–4.23)**	**0.002**	1.07 (0.53–2.14)	0.86
Psychotic Mood Disorders						
Model 1^c^	0.30 (0.04–2.16)	0.23	0.66 (0.09–4.82)	0.68	0 (NA)	0.97
Model 2^d^	0.32 (0.04–2.33)	0.26	0.73 (0.10–5.46)	0.76	0 (NA)	0.97
Non-Psychotic Mood Disorders						
Model 1^c^	**1.87 (1.20–2.89)**	**0.01**	**2.44 (1.38–4.34)**	**0.002**	1.36 (0.69–2.69)	0.38
Model 2^d^	**2.02 (1.28–3.19)**	**0.002**	**2.91 (1.61–5.27)**	**<0.001**	1.35 (0.67–2.74)	0.40
Anxiety Disorders						
Model 1^c^	0.63 (0.26–1.54)	0.31	0.57 (0.14–2.33)	0.44	0.68 (0.21–2.18)	0.52
Model 2^d^	0.62 (0.25–1.55)	0.31	0.54 (0.13–2.25)	0.40	0.71 (0.22–2.30)	0.56
Personality Disorders						
Model 1^c^	1.06 (0.43–2.61)	0.90	1.09 (0.26–4.48)	0.91	1.05 (0.33–3.37)	0.94
Model 2^d^	1.09 (0.43–2.75)	0.86	1.10 (0.26–4.68)	0.89	1.08 (0.32–3.61)	0.90
Suicides						
Model 1^c^	0.30 (0.04–2.20)	0.24	0 (NA)	0.98	0.39 (0.05–2.81)	0.35
Model 2^d^	0.31 (0.04–2.29)	0.25	0 (NA)	0.99	0.38 (0.05–2.83)	0.35
Suicide Attempts						
Model 1^c^	**3.34 (1.32–8.47)**	**0.01**	**3.84 (1.15–12.86)**	**0.03**	2.82 (0.65–12.17)	0.16
Model 2^d^	**3.94 (1.49–10.39)**	**0.01**	**5.05 (1.44–17.73)**	**0.01**	2.95 (0.64–13.60)	0.17
Organic Dementias						
Model 1^c^	0.93 (0.29–2.98)	0.91	0.73 (0.10–5.40)	0.76	1.07 (0.25–4.45)	0.93
Model 2^d^	1.09 (0.34–3.55)	0.88	0.79 (0.10–6.05)	0.82	1.32 (0.31–5.62)	0.70

a HR = Hazard Ratio.

b CI = Confidence Interval.

c Stratified for sex and year of birth and adjusted for mother’s and father’s highest attained occupational statuses, birth weight of the offspring, and maternal body mass index at childbirth.

d Stratified for sex and year of birth and adjusted for mother’s and father’s highest attained occupational statuses, birth weight of the offspring, maternal body mass index at childbirth, and maternal age at childbirth.

Sex-specific analyses showed that among women, maternal grand multiparity was associated with significantly increased, over 1.7-, 2.5-, 2.4-, 2.0-, 2.4-, and 3.8-fold risks of any severe mental disorder, non-psychotic substance use disorders, schizophrenia, schizotypal, and delusional disorders, mood disorders and particularly non-psychotic mood disorders and suicide attempts, respectively (Table 4). All these associations remained significant also after adjusting for maternal age at childbirth.

Among men, maternal grand multiparity showed no significant associations with any severe mental disorder or the specific mental disorders, suicides or suicide attempts, or with organic dementias (Table 4). Furthermore, analyses of interaction by sex showed that maternal grand multiparity interacted significantly with the sex of the offspring in predicting the risk of any severe mental disorder (p = 0.04). However, there were no significant interactions by sex in the analyses of specific types of psychopathology (all p-values≥0.07).

## Discussion

In this longitudinal cohort study, maternal grand multiparity predicted significantly increased risks of mood disorders (particularly non-psychotic mood disorders) and suicide attempts in adulthood. All these effects were stronger among women, for whom maternal grand multiparity also predicted increased risks of any severe mental disorder and of non-psychotic substance use disorders, and schizophrenia, schizotypal, and delusional disorders. The effects of maternal grand multiparity on psychopathology risk in the offspring were not secondary to maternal age at childbirth. These effects were not either explained by socioeconomic position in childhood, maternal BMI at childbirth, or by the birth weight or the birth year of the offspring. Among men, no significant associations between maternal grand multiparity and offspring risks of mental disorders were found.

Our findings show some correspondence with those of the Northern Finland Birth Cohort 1966 (NFBC), where, in a population-based study of among approximately 11000 participants, maternal grand multiparity predicted increased risks of depression, psychotic disorders and alcoholism from birth up to 28 years of age [2] and the risk of schizophrenia up to 40 years of age [3]. The NFBC study found no effects of grand multiparity on suicide attempts [1]. Yet, other large population-based studies with follow-ups until approximately 30–40 years of age have, rather in correspondence with our findings, shown that the risks of suicide attempts [4], [6] and of psychiatric hospitalizations for any reason [4] in young adulthood increase linearly with increasing maternal parity, and that these increased risks are particularly evident among offspring born as fourth or later-born children [4], [6]. Previously grand multiparity [1] and higher parity linearly and particularly as being born as a fourth-or later-born child [4]–[5] have also been shown to predict an increased risk of suicide, and third or later-born offspring have been shown to have an increased risk of personality disorders [7]. In contrast, we found no effects of maternal grand multiparity on the risks of suicides or personality disorders. While ours and the previous findings do not thus fully correspond with each other, either grand multiparity or high parity repeatedly emerges as a risk factor for severe psychopathology in the offspring.

Our study with its longest follow-up in the study field so far adds to the previous literature by suggesting that the predisposing effects of maternal grand multiparity on offspring risks of mental disorders are likely to persist across the lifespan. Our study also highlights that the effects of grand multiparity were independent of maternal age at childbirth; a finding in line with one previous study on maternal grand multiparity and schizophrenia [3]. However, confounding by maternal age has not been taken into account in other earlier studies on maternal grand multiparity and offspring risk of psychopathology [1]–[2], limiting the possibility to compare the current findings with previous ones.

We found that grand multiparity had more evident effects on the risks of severe mental disorders among women offspring. In contrast, we found no significant effects among men. In the NFBC, maternal grand multiparity had a significant effect on suicide risk only among men [1], but the previous studies on maternal grand multiparity and other diagnostic outcomes than suicides or suicide attempts did not assess moderation of effects by sex [2]–[3]. Together these findings suggest that while among men maternal grand multiparity predicts psychopathology risk only until young adulthood, among women offspring, significant effects are seen throughout the adult years. However, we found a significant interaction by sex for maternal grand multiparity only in predicting the risk of any severe mental disorder; the diagnostic category with the highest number of cases. Therefore, the sex-specificity of our findings must be interpreted with caution.

Increased levels of pre- and perinatal developmental adversities among the offspring of grand multiparous mothers offer one possible explanation for our findings of increased psychopathology risk among female offspring of grand multiparous women. Grand multiparous mothers have increased risks of gestational diabetes [12]–[13] and substance use during pregnancy [13] and more abruptions in placental functioning [11], [26]. Maternal substance use during pregnancy [9], gestational diabetes [14], and placental abnormalities [27] are each associated with increased risks of mental disorders. As stated, we controlled for maternal BMI that is associated with the risk of gestational diabetes [28], which led to no changes in the significant findings. However, we had no direct data on any of the aforementioned exposures. Since neither the previous studies on maternal grand multiparity and offspring psychopathology risk assessed confounding by these factors [1]–[3], whether or not these pre-and perinatal adversities played a contributory role to our findings remains unsolved.

Possible psychosocial explanations for our findings include exposure to compromised parenting or to early socioeconomic adversity among the offspring born to grand multiparous mothers. The offspring of grand multiparous mothers have been shown to receive less optimal parenting [29]. Also socioeconomic adversities are more common in the families with grand multiparous mothers [2], [10]. Compromised parenting [30]–[31] and early socioeconomic adversity [5], [9] have both been shown to predict increased risks of mental disorders. Although we controlled for parental occupational attainments as proxies of socioeconomic position in childhood, residual confounding by socioeconomic adversity or mediation via parenting differences remains a possibility.

By identifying a risk group for developing psychopathology later in life, the offspring and especially the women born to grand multiparous mothers, our findings may inform the planning and the targeting of early preventive interventions for mental disorders. Preventive intervention studies among another risk group for mental disorders, children born preterm [6]–[7], have suggested significant benefits of preventive interventions in reducing later risk of psychopathology [32]–[33]. Among other things, these interventions have focused on improving parenting practices in the families of preterm children [32]–[33]. Since children born to grand multiparous mothers receive less optimal parenting [29], they constitute another target group that may benefit from preventive interventions, focusing, for example, on improving parenting practices.

The strengths of our study include the birth record data on parity and the long diagnostic follow-up allowing us to examine the effects of maternal grand multiparity on mental disorders from early to late adulthood. Overall, the diagnostic data in the HDR and CDR are validated and suitable for research [15]–[23], and of mental disorders, especially the diagnoses of affective and non-affective psychotic disorders are well validated [18]–[20]. However, there are limitations to our study. We are unaware of any studies on the validity of the HDR or CDR diagnoses on anxiety or personality disorders, or on suicides or suicide attempts. The validity of these diagnoses in the registers thus remains to be proven. Extracting data on mental disorders diagnoses only from the HDR and CDR means that our findings generalize best to severe mental disorders. Using only these registers misses the outpatient diagnosis and the generalizability of our findings to less severe psychopathology is limited. Anxiety and personality disorders that are most often treated in outpatient clinics may have less comprehensive coverage in these registers than the other diagnostic outcomes.

Furthermore, we had diagnostic data available only from 1969 onwards. Hence, we were unable to identify those individuals who were hospitalized or who had died with mental disorders before this year when the participants were between 24 to 34 years old, and who were not hospitalized with mental disorders again thereafter. There are hence some false negatives in our sample: participants who have had severe mental disorders early but not later in adulthood. Due to these limitations, our findings best generalize to severe mental disorders diagnosed between the ages 24–34 and 65–76. We have no data on gestational diabetes, placental abnormalities, maternal smoking, the quality of parenting, on family structure later in childhood, or on societal support the families may have received and could not thus assess whether these were underlying contributory factors for our findings. We were also unable to assess possible confounding by paternal age or by parental psychopathology, both of which are associated with grand multiparity [2]–[3] and predict psychopathology risk in the offspring [1], [3], [6], [8]–[9], and may hence have mediated or moderated our findings. However, previous studies have suggested that the effects of maternal grand multiparity on offspring psychopathology risk occur independently of parental psychopathology [1]–[2]. Since suicide attempts were encoded to the HDR only until 1986, the diagnostic follow-up for them was shorter than for the other diagnoses although still extending to when the participants were between 42 and 52 years of age. Generally, the two changes in the diagnostic classification system during the diagnostic follow-up might have created heterogeneity to the diagnostic groups. Finally, when interpreting our findings, one must consider the specific living circumstances in Finland during World War II, the time period when our cohort members were born and spent their early childhood. During the war, bombings, poverty, and malnutrition were very common in Finland, fathers were often away from home at the battlefront and many children were evacuated to interim foster care abroad. The circumstances were thus specific in many ways, which may limit the generalizability of our findings to individuals born more recently. Nevertheless, especially among women, our findings were comparable to those in younger cohorts.

In conclusion, especially among women offspring, maternal grand multiparity had widespread long-term effects on the risks of severe mental disorders that seemed to persist across the lifespan, while among men, no such effects were found. Our findings highlight maternal grand multiparity as an emerging risk factor for severe mental disorders, and may enable identification of high-risk individuals that may benefit from targeted preventive interventions before the occurrence of severe mental health problems.
